# Highly selective and flexible silver nanoparticles-based paper sensor for on-site colorimetric detection of paraquat pesticide†

**DOI:** 10.1039/d4ra04557b

**Published:** 2024-09-10

**Authors:** Sanjeev Bhandari, Vijay Singh Parihar, Minna Kellomäki, Mrityunjoy Mahato

**Affiliations:** a Physics Division, Department of Basic Sciences and Social Sciences, School of Technology, North-Eastern Hill University Shillong Meghalaya 793022 India mrityunjoyphy1@gmail.com; b Biomaterials and Tissue Engineering Group, Faculty of Medicine and Health Technology, Tampere University 33720 Tampere Finland vijay.parihar@tuni.fi

## Abstract

Paper-based sensors or paper-based analytical devices (PADs) have recently emerged as the cost-efficient, and portable, on-site detection tools for various biological and environmental analytes. However, paper-based sensors often suffer from poor selectivity. Here, a single-step paper-based flexible sensor platform has been developed for the on-site detection of paraquat (PQ) pesticide in real samples, utilizing chitosan and citrate-capped silver nanoparticles integrated with a flexible paper. The nanocomposite paper film was thoroughly characterized using UV-visible spectroscopy, Fourier-transform infrared spectroscopy (FTIR), and transmission electron microscopy (TEM). The composite paper platform demonstrated a color change with a reaction time within a few minutes (6–7 min) in the presence of PQ pesticide. The trace level PQ pesticide has been detected with a limit of detection (LOD) of 10 μM and a linear range (LR) of 10–100 μM. The sensor shows 3× more selective signal towards PQ pesticide compared to other similar pesticides. The relative standard deviation (RSD) was found to be 5% for repeatability, 4% for reproducibility, 2% for interference, and 3.5% for real sample analysis, indicating high precision sensing and within the WHO limit of RSD (20%). The present work will open up new avenues for the advancements in flexible paper sensors; cost-effective, portable, on-site sensors, and sustainable device development.

## Abstract 2

Paper-based sensors or paper-based analytical devices (PADs) have recently emerged as the cost-efficient, and portable, on-site detection tools for various biological and environmental analytes.

## Graphical abstract

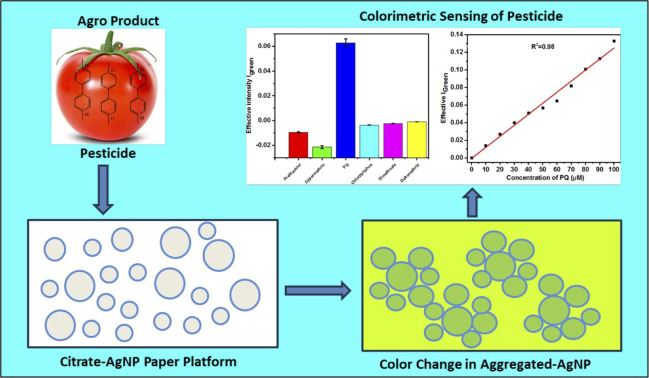

## 1. Introduction

Chemical pesticides have demonstrated their potential since the 1940s by increasing global agricultural productivity, reducing insect-borne diseases, and protecting wood products.^1,2^ However, the amount of pesticide reaching the target pest is 0.1% only, and the remaining 99.9% goes into the environment, water bodies and the food chain.^2^ Pesticides are non-degradable toxic chemicals, which can lead to diseases such as kidney dysfunction, birth defects, neuro disorder, and cancer.^2,3^ On the other hand, 50% of the world population directly depends on the agriculture profession and almost the entire global population indirectly depends on agriculture.^3^ Hence, pesticide detection, control use and pesticide destruction are some of the urgent issues in the agro sector.

In this regard, paper-based sensors, also known as paper based analytical devices (PAD), present a cost-effective, rapid detection solution for pesticide in agro field settings, due to the cheap cellulose, controlled porosity, and biocompatibility.^3,4^ Paper sensors need specific recognition elements to be added, such as enzymes, nanoparticles, and molecularly imprinted polymers (MIPs), to detect target pesticides in real samples.^5–7^ Paper based methods include colorimetry, fluorescence, surface enhancement Raman scattering (SERS), and electrochemical method, where the majority of techniques are time-consuming, costly and require complicated instrumentation.^4^ Among these, paper based colorimetric methods provide naked eye observation or by mobile based image analysis and without any sophisticated instruments. The colorimetric method mostly relies on the aggregation phenomena of nanoparticles, which leads to the color modulation of the nanoparticle on the sensing platform.

Silver nanoparticles (AgNPs) have become popular for colorimetric sensors,^8^ due to their size, shape, interparticle distance and functionalization dependent optical properties and also due to high surface energy or surface reactivity.^9–11^ AgNPs can be functionalized with specific receptors or ligands that selectively bind to target pesticide molecules, enabling the development of highly sensitive and selective sensors.^8^ AgNPs have potential in colorimetric sensors due to their aggregation properties through interaction with the analyte.^12^ The aggregation phenomena of AgNPs can be further tailored by capping agent and through its surface plasmon resonance phenomena.^12^ Dubas *et al.* synthesized poly(methacrylic acid)-AgNP and used it for colorimetric sensing of ammonia.^13^ Orouji *et al.* used uncapped AgNP at different pH for aggregation induced detection of organophosphorus pesticide.^14^ Jana *et al.* synthesized silver-carbon dot hybrid and used it for sensing of ethanol.^15^

Paraquat (1,1-dimethyl-4,4-pyridinium chloride, PQ) has been used worldwide as herbicide in agriculture since its discovery and after its commercial approval in 1955.^1,16^ Paraquat ions bind to the ferredoxin binding site of photosystems, destroying cell membranes and ultimately causing the death of the plant.^1^ The estimated oral lethal dose (LD) of paraquat in humans is 35 mg kg^−1^.^17^ PQ has long-term persistence and is toxic causing damage in brain, lungs, liver, and kidneys.^18^ Hence, analyzing herbicide quantitatively with a portable, low-cost, high-sensitive sensor is essential.

In literature, several attempts have been made to detect the PQ by various approaches, including spectroscopic,^19^ fluorimetric,^20^ electrochemical,^21^ and colorimetric,^22^ liquid chromatography/electrospray ionization-mass spectrometry, ultraperformance liquid chromatography-mass spectrometry/mass spectrometry (UPLC-MS/MS).^23^ Traiwatcharanon *et al.* prepared the PbO-NPs/SPE for sensing of PQ herbicide with a linear range of 1–5 mM using electrochemical technique (with PbO-NPs as an active compound).^24^ Somnet *et al.* prepared platinum nanoparticles coated with a molecularly imprinted polymer (PtNPs@MIP) for sensing PQ with a detection limit of 20 mM using electrochemical technique.^25^ Zhao *et al.* prepared a pyranine-based fluorescent “turn-off” method for PQ sensing with a linear range of 1–20 μM.^26^ Kong *et al.* prepared AuNP–GO composite for sensing of PQ using electrochemical method.^27^ Xiong *et al.* prepared a carboxyl group functionalized AuNP for electrochemical detection of PQ pesticide.^28^ Shan *et al.* prepared Au–chitosan composite for electrochemical detection of PQ pesticide.^29^ Chang *et al.* fabricated a paper-based and image analysis based sensor for sensing PQ with a detection limit of 28 μM.^30^ Chaikhan *et al.* designed a PQ sensor with chromatography paper with a detection limit of 1.24 mg L^−1^.^31^ The PAD-based sensors are more costly than paper-based sensors due to the use of inkjet printing and wax printing. Wang *et al.* reported a paper sensor modified by mesoporous silica coupled with carboxylatopillar[5]arene for PQ detection, however used surface enhanced Raman scattering (SERS) method, which is costly and not portable.^32^ To the best of our knowledge, there is no colorimetric paper-based PQ pesticide sensor has been reported in the literature. Also the existing PQ sensor rarely addressed sensing parameters like selectivity, interference and repeatability.^30,31^ The paper based pesticide sensors are struggling with selectivity issues, which have been addressed in the present work.

We have prepared a paper-based colorimetric sensor consisting of citrate-capped silver nanoparticles (cc-AgNPs) and chitosan for selective detection of PQ herbicide. Chitosan has been selected as a robust, biocompatible matrix for developing a sensing platform due to its film-forming ability through its cationic amino group and chelating property to metal ions.^33,34^ The nanocomposite paper film has been thoroughly characterized using UV-visible spectroscopy, Fourier-transform infrared spectroscopy (FTIR), and transmission electron microscopy (TEM). The selectivity for paraquat pesticide was attained using the modified paper-based sensor, with a limit of detection (LOD) and linear range (LR) of 10 μM and 10–100 μM, respectively. The other sensor parameters, such as reproducibility, interference, and real samples analysis, showed the RSD% values as 5%, 2%, and 3.5%, respectively, which are within the WHO recommendation of 20%. The present work will open up new avenues for advancement in flexible electronics; cost-effective, portable and on-site sensors, and sustainable device development.

## 2. Experimental method

### 2.1 Materials

Chitosan (CS), ascorbic acid, and trisodium citrate were purchased from Sigma-Aldrich, USA. Silver nitrate (AgNO_3_), sodium hydroxide (NaOH), and acetic acid were purchased from SRL, India. Chlorpyrifos, pretilachlor, cypermethrin, paraquat, deltamethrin, and dimethoate were purchased from Shillong, India. Deionized water with pH = 6.5 and resistivity = 18.5 MΩ × cm, was used for making solutions for different pesticides.

### 2.2 Preparation of citrate-capped silver nanoparticles (cc-AgNPs) and chitosan solution (CS)

**Fig. 1:**
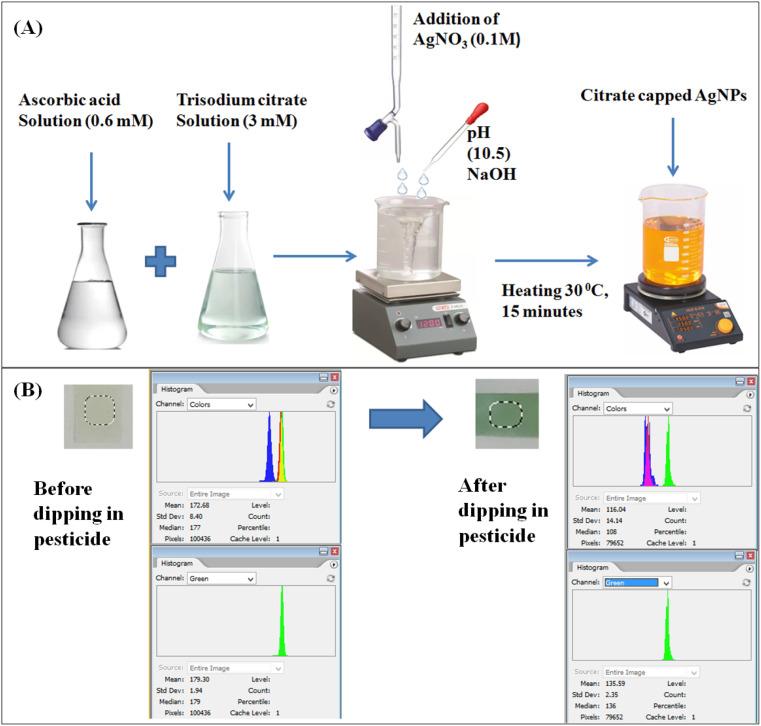
(A) Flow chart for preparation of citrate-capped silver nanoparticles. (B) Working mechanism for citrate-capped AgNPs paper-based pesticide sensor.

Citrate-capped silver nanoparticles (cc-AgNPs) were prepared using the procedure by Alula *et al.*^12^ Firstly, ascorbic acid (0.6 mM) and trisodium citrate (3 mM) were prepared in deionized water. These two solutions were mixed in the conical flask, and the pH (10.5) was adjusted using NaOH solution (0.1 M) under continuous stirring. Later, AgNO_3_ solution (0.1 M) was added to the conical flask containing the two solutions. The solution was heated to 30 °C for 15 minutes, resulting in a yellow color solution. The yellow solution indicates the preparation of the cc-AgNPs colloids, as shown in Fig. 1A. The CS solution with a concentration of 0.1 M was prepared by mixing chitosan flakes with acetic acid (0.1 M) under constant stirring for 6 hours at room temperature.

### 2.3 Smartphone camera and imaging characterization

All images of the paper discs were taken by the smartphone camera. Smartphone model name (Redme Note 8 pro), camera pixel (64 × 10^6^), camera F stop (f/1.9), color representation (RGB), and object distance (32 cm). Modified paper surfaces have been characterized with FTIR (model) and TEM (model).

### 2.4 Preparation of paper-based sensor and its measurement

Firstly, Whatman filter paper (44) was immersed in the chitosan solution for 15 seconds and allowed to vacuum dry overnight. In the second stage, the chitosan-attached Whatman filter paper (44) was immersed in the citrate-capped silver nanoparticle solution for 15 seconds and vacuum-dried overnight. The prepared paper-based sensor was used to sense paraquat (PQ) pesticides for further studies.

The detection of PQ relies on the reaction between the CS + cc-AgNPs probe and PQ pesticide. For this purpose, varying concentrations of PQ was treated with the designed paper probe and allowed to react for 9 min. A smartphone camera recorded the color changes on paper discs in photographic conditions. This was followed by image processing and colorimetric quantification, where the colored images of the paper surface were processed to obtain the corresponding RGB values. A uniform selective area was adapted for the image analysis to eliminate the errors in pixel values in the capturing condition. The selective area eliminates the dark rings and patches. It provides accurate changes in the pixel values by taking mean intensity values of the distributed uniform area of the CS + cc-AgNPs paper-based sensor. Fig. 1B shows a change in RGB intensity when CS + cc AgNPs interact with PQ pesticide.

## 3. Results and discussion

### 3.1 FTIR and TEM characterization of citrate-AgNP based paper sensor platform

**Fig. 2:**
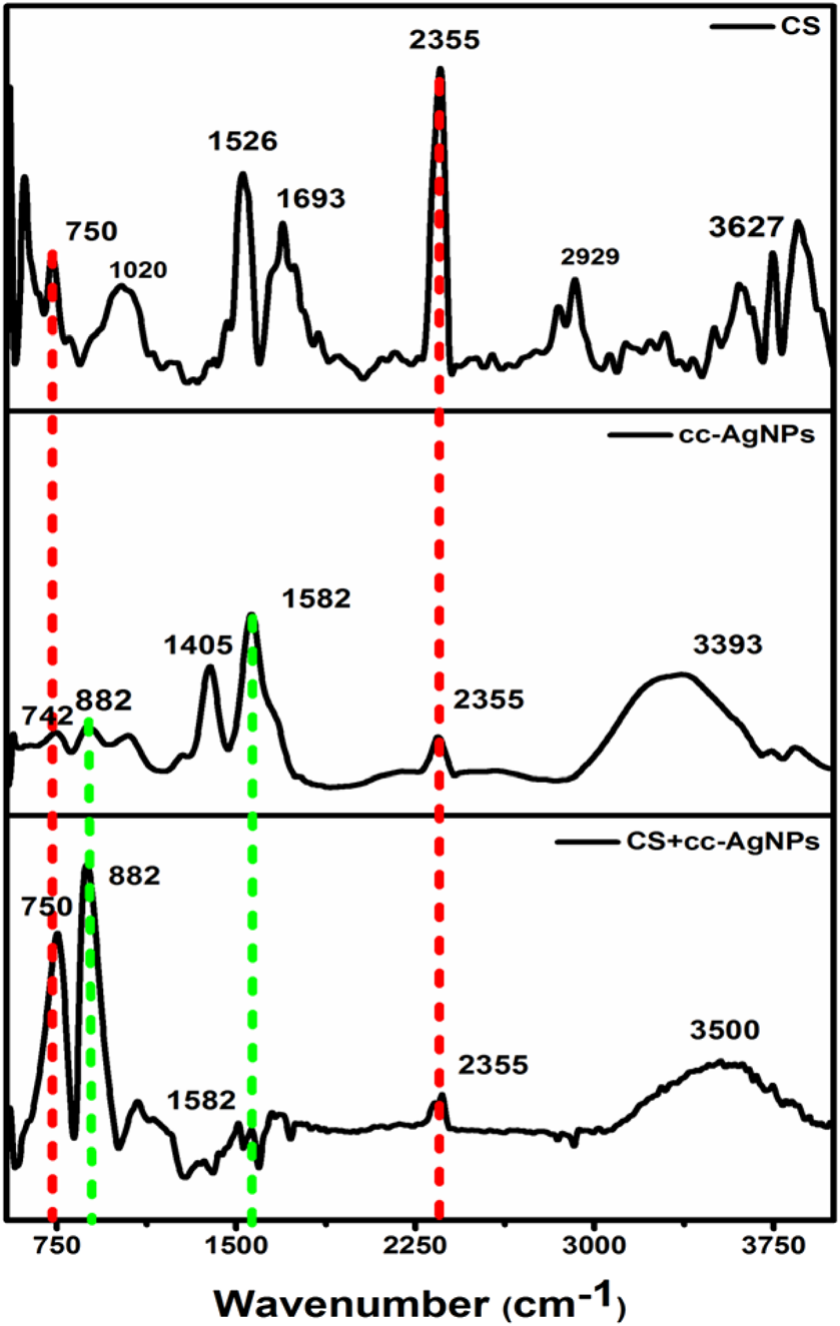
FTIR graph of components of nanocomposite and nanocomposite film.

FTIR spectra of the chitosan (CS), cc-AgNPs, and, CS + cc-AgNPs are shown in Fig. 2. The characteristic peaks of chitosan are present at 1020 cm^−1^ (C–O–C stretching vibrations) due to an ester group of CS.^33^ The band at 2920 cm^−1^, 1526 cm^−1^, and 750 cm^−1^ corresponds to the C–H stretching, amide III, and N–H bending vibration of chitosan, respectively.^33,35,36^ Also, the band at 3627 cm^−1^ corresponds to the O–H stretching vibration in chitosan.^37^ For cc-AgNPs, the presence of bands at 1582 cm^−1^ and 1405 cm^−1^ correspond to the asymmetric and symmetric C=O stretch of carboxylate ion, respectively, and a wide band at 3393 cm^−1^ corresponding to the O–H stretch proves the surface capping of trisodium citrate on silver nanoparticles.^38^ A band at 882 cm^−1^ is present due to the stretching vibration of the C=O bond in the carboxylate group of the citrate ligand.^39^ Also, the band at 2355 cm^−1^ is due to C–N stretching.^40^ The shift from 3393 cm^−1^ to 3350 cm^−1^ can be attributed to the interaction between citrate-capped silver nanoparticles and chitosan through hydrogen bonding, leading to a change in the hydrogen bonding environment of the hydroxyl groups. Peak assignments of the composite film are shown in Table S1.†

**Fig. 3:**
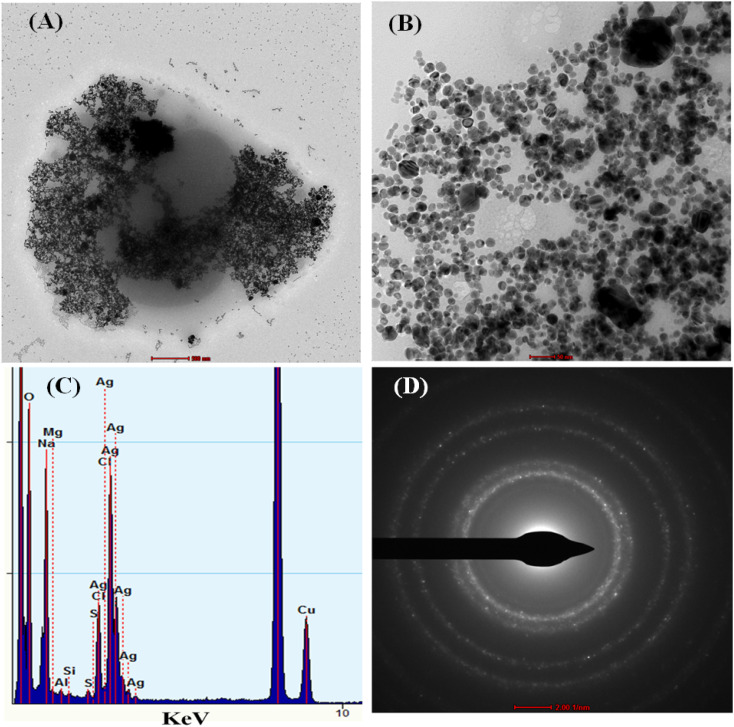
TEM characterization of cc-AgNP with PQ, (A and B) TEM images, (C and D) EDX analysis, and SAED pattern.

The TEM characterization has also been performed to study the pesticide interaction with cc-AgNPs. Fig. 3A and B show the TEM image of cc-AgNPs with PQ pesticide on a nanometer scale. When PQ (10 μM) was mixed with the cc-AgNPs solution, the aggregation of the cc-AgNPs took place, as shown in Fig. 3A. The interaction may be due to chemical groups in pesticides that interact with the surface of the nanoparticles.^41^ This interaction could lead to the formation of chemical bonds or attractive forces that cause the nanoparticles to aggregate. The energy dispersive X-ray analysis (EDX) shows the elemental analysis of the sample (Fig. 3C), which confirms more than 60% Ag atoms from AgNP, Cu atom due to the TEM grid, and the presence of other elements (Fe, Al) in low% is due to spurious X-rays.^42^ The d-value from the SAED pattern (Fig. 3D) is calculated for different planes ((111), (200), (220), (311)) as 0.24 nm, 0.19 nm, 0.14 nm, 0.12 nm, respectively, which aligns with the literature report.^41,43,44^

### 3.2 Optimization of paper type, pH, and reaction time for sensing

**Fig. 4:**
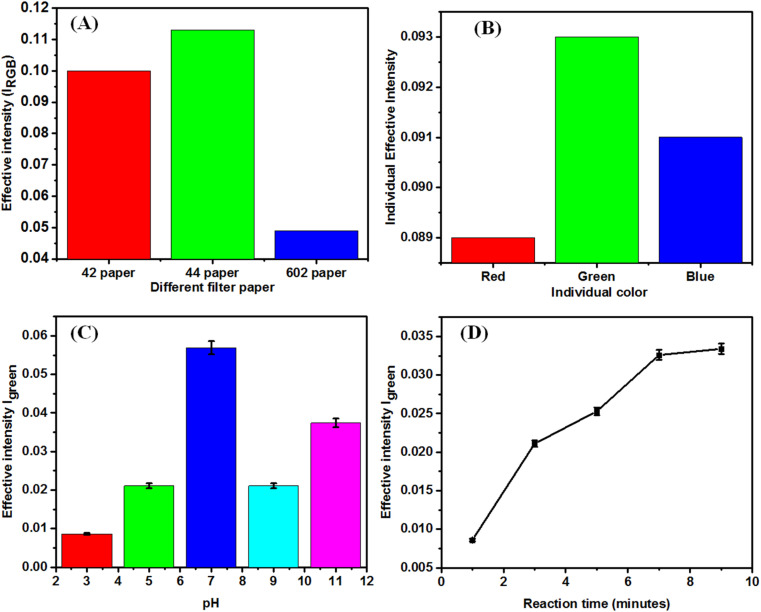
(A) Selection of the paper using RGB intensity and (B) the selection of the individual intensity (30 μM). (C) Graph showing the signal intensity change with the reaction time (selected for pH 7, 30 μM). (D) Graph showing the signal intensity change with the reaction time (selected for 9 minutes, 30 μM).

We have spread the nanocomposite on the three different Whatman papers (42, 44, and 602). We checked color intensity of individual paper and Whatman (44) was optimized (Fig. 4A). The green intensity was found to be the highest, which was chosen for colorimetric sensing compared to other colors (red and blue) for all studies (Fig. 4B). To find out the color channel that provides maximum sensitivity for the analytical applications of the designed biosensor, we individually tested and compared the primary color channels.

The pH study was carried out by varying the pH from 3 to 11. Fig. 4C shows the signal intensity variation with the different pH. The pH of the test solution was adjusted using HCl or NaOH. The concentration used for the study was 30 μM of PQ pesticide. Maximum responses were obtained at pH = 7, and the response decreased on acidic and alkaline pH (Fig. 4C). The prepared paper-based sensor was tested for different reaction times, and it was found that it gradually increased and got saturated and optimized after 9 min of reaction time for sensing pesticides (Fig. 4D). Water contact angle measurements were taken to assess the hydrophobicity and water absorption on Whatman paper, chitosan-modified paper, and CS + cc-AgNPs-modified paper as shown in Fig. S1.† While no contact angle was measurable for Whatman paper due to strong liquid–solid attraction, chitosan-modified paper showed a hydrophobic contact angle of 109°, and CS + cc-AgNPs-modified paper had a contact angle of 116°, indicating it is nearing superhydrophobicity.

### 3.3 Detection of PQ using the CS + cc-AgNPs paper-based sensor

To evaluate the colorimetric sensor, the RGB (red, green, and blue) convention of color image analysis was followed, where we first selected the most sensitive primary color channel, as seen in Fig. 4A. The effective intensity was calculated using eqn (1).^45^1
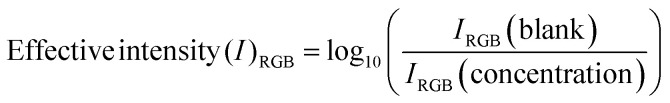


*I*_RGB_ is the mean pixel intensities of all primary channels obtained from a selected area. In digital images, the intensity *I*_RGB_ reflects pixel intensity of the actual color, composed of the intensities of three primary color channels: *I*_Red_, *I*_Green_, and *I*_Blue_. In the colorimetric changes, gradual intensification of the appeared color signifies the absorption of the complementary color.^46^ To find out the color channel that provides maximum sensitivity for the analytical applications of the designed biosensor, we individually tested and compared the primary color channels as seen in Fig. 4B. The individual intensity was calculated using eqn (2).^45^ The *I*_Green_ was selected for all the further studies.2
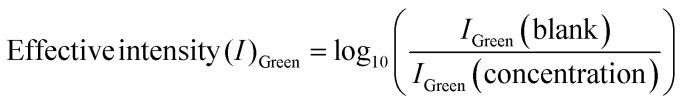
where, *I*_Green_ is the mean pixel intensity of the green channel obtained from the selected area.

### 3.4 UV-visible spectra of CS + cc-AgNPs and selectivity study

**Fig. 5:**
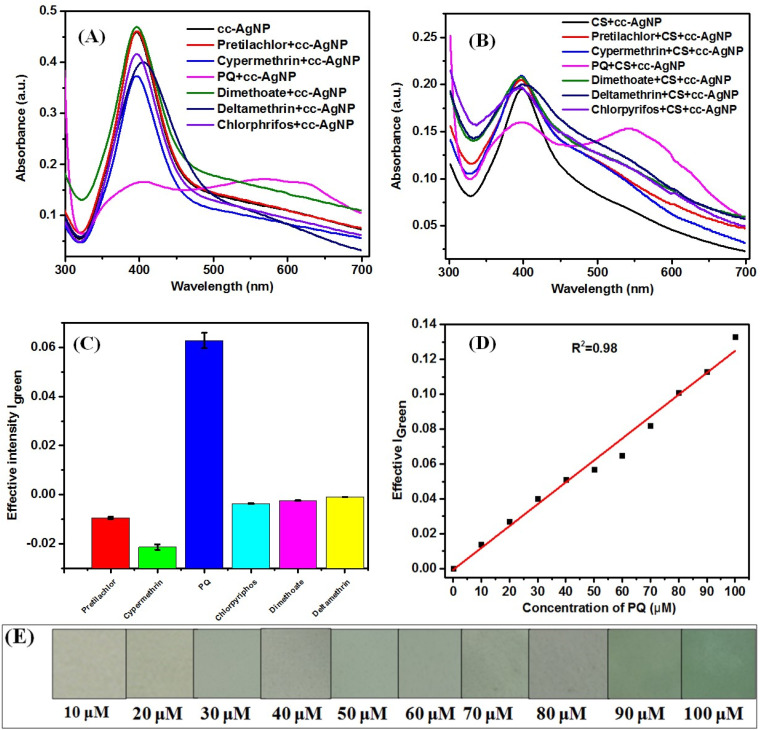
(A) UV-visible spectra of cc-AgNP with different pesticides, (B) UV-visible spectra of CS + cc-AgNP with different pesticides, (C) selectivity of the prepared CS + cc-AgNPs paper-based sensor, (D) calibration curve of the prepared paper-based sensor, (E) color changes recorded of the CS + cc-AgNP modified paper at different concentration of PQ pesticide.

Fig. 5 shows the UV-visible spectra of cc-AgNPs and CS + cc-AgNPs in the presence of different pesticides, which shows an intense peak around 405 nm due to localized surface plasmon resonance (LSPR) band of metallic AgNP (Fig. 5A).^18^Fig. 5A and B indicate that the intensity of the 405 nm peak varied by adding different pesticides. However, a significant decrease in intensity was observed when PQ pesticide was added (Fig. 5A and B), which reflects a reduction in the concentration of individually dispersed cc-AgNPs in suspension and the red shift in the LSPR band (550 nm) reflects the increase of particle size or aggregation of cc-AgNP.^18,47^ Citrate (trisodium citrate) acts as a stabilizing agent, capping agent to AgNPs to prevent aggregation in normal condition and provide a negatively charged surface.^48^ Paraquat, a positively charged pesticide^49^ and hence can interact electrostatically with the negatively charged cc-AgNPs. The chitosan matrix may absorb paraquat due to its porous structure and it can make hydrogen bonding with paraquat through amine groups.^50^ The schematic diagram for the sensing mechanism or interaction of PQ pesticide with active platform, have been presented in Fig. S2.† The interaction of CS + cc-AgNP with paraquat, facilitates the aggregation of AgNP leading to color change and change in their localized surface plasmon resonance (LSPR) detectable by UV-visible spectroscopy or even by the naked eye.^47^ These types of intercation of PQ with the CS + cc-AgNP paper based platform, bring the selectivity of PQ sensing.

Fig. 5C shows the selectivity study of the PQ pesticide carried out by paper-based colorimetric measurements along with other pesticides such as pretilachlor, cypermethrin, PQ, chlorpyriphos, dimethoate, and deltamethrin. It was found that the effective intensity in presence of PQ was 3× higher signal compared to other pesticides. Therefore, PQ showed better selectivity and was selected as a target sensor analyte for determining parameters like linear range, detection limit, interference and real sample studies.

Fig. 5D shows the calibration curve of the prepared paper-based sensor with different concentrations of PQ pesticides. The limit of detection (LOD) has been calculated using the calibration curve's slope and standard deviation (SD). The LOD was determined using eqn (3) (Fig. 5D)^33^ and it was found to be 10 μM using a paper-based sensor. The LR value of a sensor is generally defined and calculated from the linear fitting of the calibration curve with *R*^2^ value > 0.95 or within a 5% deviation. The linear fitting of the signal response with a concentration of PQ pesticide has been done and LR value of the sensor have been found out to be 10–100 μM with the *R*^2^ value of fitting as 0.98. Fig. 5E shows the colour change of the CS + cc-AgNPs film after adding different amounts of PQ pesticides. Table 1 summarizes the available literature on PQ pesticide sensing using different composite and different techniques.3
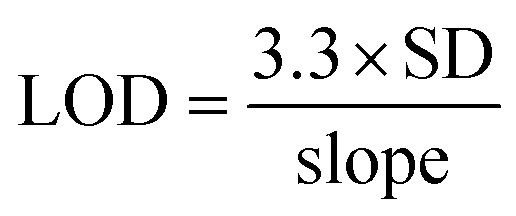


**Table 1:** Summary of PQ pesticide sensing parameters from literature data and present work

S. No.	Nanocomposite used	Technique	LOD	Linear range	Sensitivity	RSD% (real sample/reproducibility)	References
1	Citrate-AgNP	Paper-based colorimetric	10 μM	10–100 μM	NR	3%/4%	Present work
2	PbO NPs/SPE	Electrochemical	1.1 mM	1–5 mM	204.85 mA mM^−1^ cm^−2^	5.95%	51
3	GCE	Electrochemical	3.2 μM	3.9–31.0 μM	NR	NR	52
4	PtNPs/MIP	Electrochemical	0.02 μM	0.05–1000 μM	NR	NR	25
5	Tb-MOF	Photoluminescence	2.84 μM	0–50 μM	NR	NR	53
6	Carboxylatopillar[5]arene-SiO_2_ paper	SERS	0.000117 μM	NR	NR	NR	32
7	Nf/SPGE	Electrochemical	0.31 μM	5.0–125 μM	NR	4.78%	54
8	SiO_2_ modified GCE electrodes	Electrochemical	12 nM	10 nM to 10 μM	0.021 μA nM^−1^ cm^−2^	NR	55
9	Graphene-B-diamond electrode	Electrochemical	10 μM	0.2 and 1.2 μM	31.83 μA μM^−1^ cm^−2^	NR	56
10	Au NPs/caboxylato-pillar[5]arenes	Electrochemical	0.73 nM	3.8–10 μM	NR	NR	57

### 3.5 Repeatability, reproducibility, interference, and real samples study

**Fig. 6:**
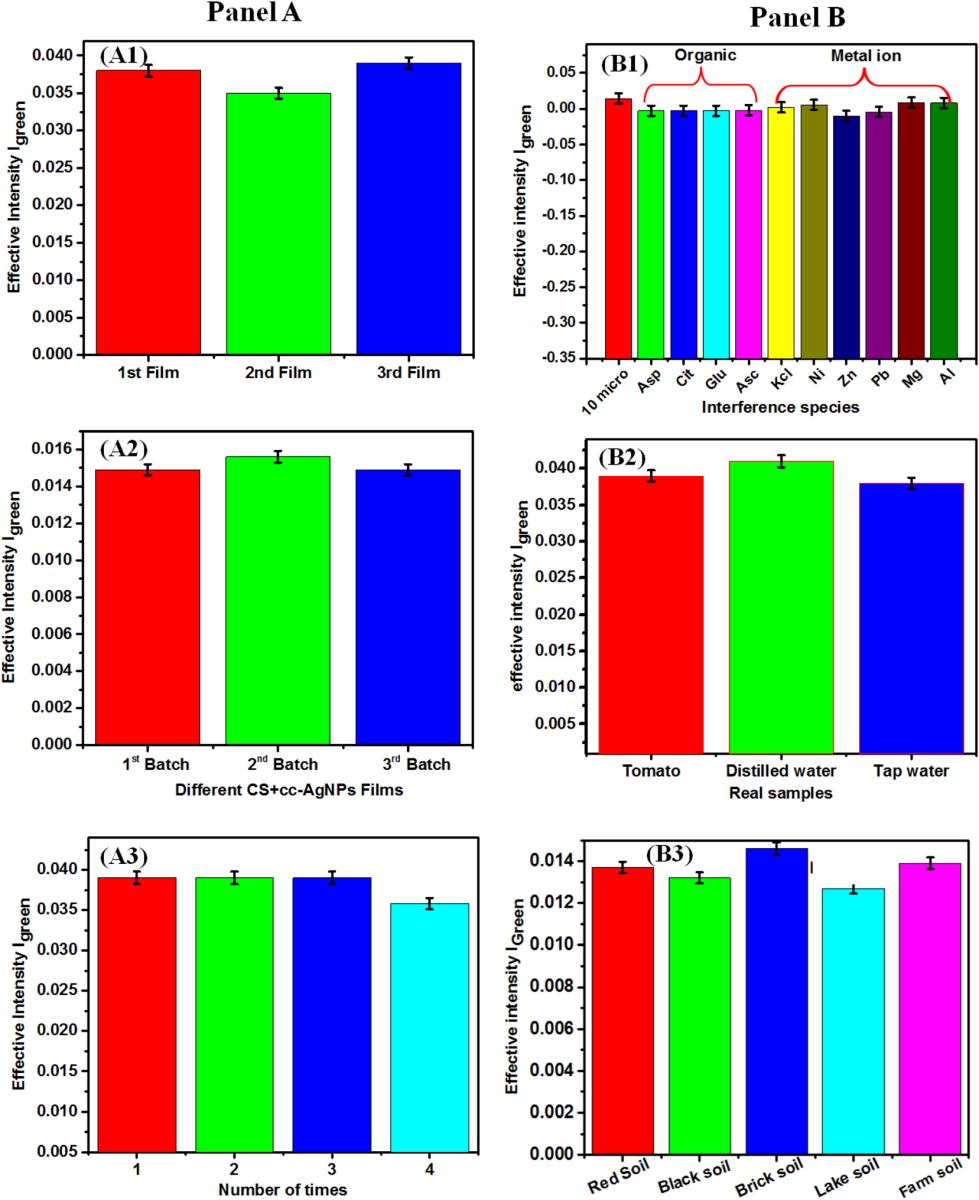
Panel-A: (A1) reproducibility study of the sensor with different CS + cc-AgNPs films in presence of PQ pesticide, (A2) reproducibility study of the sensor with different CS + cc-AgNPs batch in absence of PQ pesticide, (A3) repeatability study of the sensor; panel-B: interference studies in different interfering species and real samples: (B1) metal ions and organic interfering species, (B2) food samples analysis, (B3) soil samples analysis.

In Fig. 6, panel A shows the reproducibility and repeatability of the paper based sensor. The reproducibility of the sensor was tested on 3 different CS + cc-AgNPs modified paper film with RSD value of 4% (Fig. 6A1). The films were prepared under the same ambient conditions and the same paper have been cut into three pieces of films. The CS + cc-AgNP paper acts as a platform for colorimetric sensing of PQ pesticide and the justification of its standardization and preparation are as follows. The cc-AgNP solution is the standard solution since it has been prepared following simple chemical mixing at a given standard condition and no further centrifugation or filtration. The paper used was Whatman paper (grade 44) and hence it is standard one. We have set the standards of the coating method of CS + cc-AgNP on paper by following protocol. To prepare CS + cc-AgNP, we have taken chitosan with standard molecular weight (MW 150–700 kDa, 90% DA) and cc-AgNP was prepared as mentioned section 2.2. The reaction time of cc-AgNP with paper has been optimized through visual inspection until the mechanical strength of the paper looses, as 15 s. The CS + cc-AgNP coating on paper was done by two steps *i.e.* 15 s dipping of paper in chitosan solution followed by overnight (12 h) dried at vacuum desiccator. Afterword, the chitosan-coated Whatman paper was dipped in cc-AgNPs for 15 s and similarly dried overnight (12 h) under vacuum. These two step methods made the coating process bias less and reproducible batch synthesis, with RSD value of reproducibility within 4.1% (Fig. 6A2). The repeatability of the reported sensor has been carried out (Fig. 6A3) using 4 numbers of paper-based sensors and the RSD value of the sensor responses was 5%, which indicates the reproducibility of the sensor.

The Fig. 6, panel-B shows the studies of interference with metal oxide, organic species, food samples and soil samples, respectively. Fig. 6B1 shows the interference studies carried out using 10 μM of PQ pesticide in the presence of 2 μM metal ions (Zn^2+^, Cu^2+^, Ni^2+^, Al^3+^, Mg^2+^) as well as 2 μM organic interfering similar molecules (ascorbic acid, aspartic acid, glutamic acid, citric acid), with the variation of signal response within RSD value of 2% which represents the consistency of sensor response in the presence of interfering species. Fig. 6B2 shows the sensor response for food samples (tomato, distilled water, and tap water), with RSD value of 3%, which shows that the paper-based sensors could be used for real sample or matrix such as food sample analysis. In order to achieve real sample analysis in more versatile manner, the interference studies with soil sample was also conducted. The soil samples (black, red, brick, lake, and farm) were collected from various surface sources following an earlier report.^58^ The collected samples were sieved and a specific quantity (10 grams) of each soil, was dissolved in 25 mL of DI water and left for 8 hours for sedimentation of the dirty layer. The samples were filtered and collected in a 50 mL volumetric flask and then spiked with PQ pesticide solution (10 μM). The scheme for preparing soil samples is shown in Fig. S3.† The pre-treatment procedure for soil samples yielded favourable recovery results as shown in Table 2, which indicates a significant improvement for practical detection of PQ in soil samples. The paper-based flexible sensor demonstrated excellent average RSD value with 3.5% for soil sample analysis. The different sensing parameters obtained from the food and soil samples indicates the on-site sensing relevance.

**Table 2:** Application of paper based sensor for the determination of PQ in spiked soil samples

S. No.	Soil samples	Spiked (μM)	Recovered (μM)	Recovery (%)
1	Farm soil	10	8.47	84.7
2	Lake soil	10	9.52	95.2
3	Black soil	10	8.75	87.5
4	Red soil	10	8.19	85.9
5	Brick soil	10	7.82	74.2

## 4. Conclusion

We have developed a straightforward, single-step paper-based flexible sensor platform composed of chitosan and citrate-capped silver nanoparticles on Whatman paper for on-site detection of PQ pesticides in real samples. The nanocomposite was thoroughly characterized using UV-visible spectroscopy, FTIR, and TEM. The colorimetric sensing signal was quantified and effectively analyzed using Adobe Photoshop, which produces the calibration curve of the sensor. The prepared paper-based flexible sensor showed the LOD and LR as 10 μM and 10–100 μM, respectively. The prepared paper-based sensor could selectively sense PQ pesticide with an effective intensity of 3× compared to other pesticides. The paper-based flexible sensor demonstrated excellent RSD value with 5% for repeatability, 4% for reproducibility, 2% for metal ions/organic interference, and 4% for food sample analysis, and 3.5% for soil sample analysis, indicating high precision sensing capability. Therefore, all sensor parameters were within the RSD value of 20% recommended by the WHO, indicating reliable and consistent performance for detecting PQ pesticides. The present work will open up new avenues for advancement in flexible electronics, cost-effective, portable, on-site sensors, and sustainable device development.

## Data availability

The datasets generated and/or analyzed during this study will be available in the Trepo, the institutional repository of Tampere University (https://trepo.tuni.fi/). Additionally, the data supporting this article have been included as part of the Main Manuscript and ESI.†

## Conflicts of interest

The authors have no conflicts of interest to declare.

## Supplementary Material

RA-014-D4RA04557B-s001
