# Supplementary material for: Highly selective and flexible silver nanoparticles-based paper sensor for on-site colorimetric detection of paraquat pesticide
Source: RSC Adv. 2024 Sep 10;14(39):28844–53. doi: 10.1039/d4ra04557b (PMC11386213; doi:10.1039/d4ra04557b)
Supplement: RA-014-D4RA04557B-s001 [file RA-014-D4RA04557B-s001.pdf]

Supplementary Information (SI) for RSC Advances.
This journal is © The Royal Society of Chemistry 2024

Supplementary Information

Highly Selective and Flexible Silver Nanoparticles-Based Paper Sensor for on-site Colorimetric Detection of Paraquat Pesticide

Sanjeev Bhandari[a], Vijay Singh Parihar[b*], Minna Kellomäki[b], Mrityunjoy Mahato[a*]

[a]Physics Division, Department of Basic Sciences and Social Sciences, School of Technology, North-Eastern Hill University, Shillong, Meghalaya, 793022, India.

[b]Biomaterials and Tissue Engineering Group, Faculty of Medicine and Health Technology, Tampere University, 33720 Tampere, Finland.

Table S1: Peak assignments of the composite film.

S.No.	Peak (cm^{-1})	Peak assignment	References
1.	1020	C-O-C stretching	34
2.	2920	C-H stretching	35
3.	750	N-H bending	33
4.	3627	O-H stretching	37
5.	1582	Asymmetric C=O stretching	38
6.	1405	Symmetric C=O stretching	39
7.	3393	O-H stretching	38
8.	882	C=O stretching	39
9.	2355	C-N stretching	40

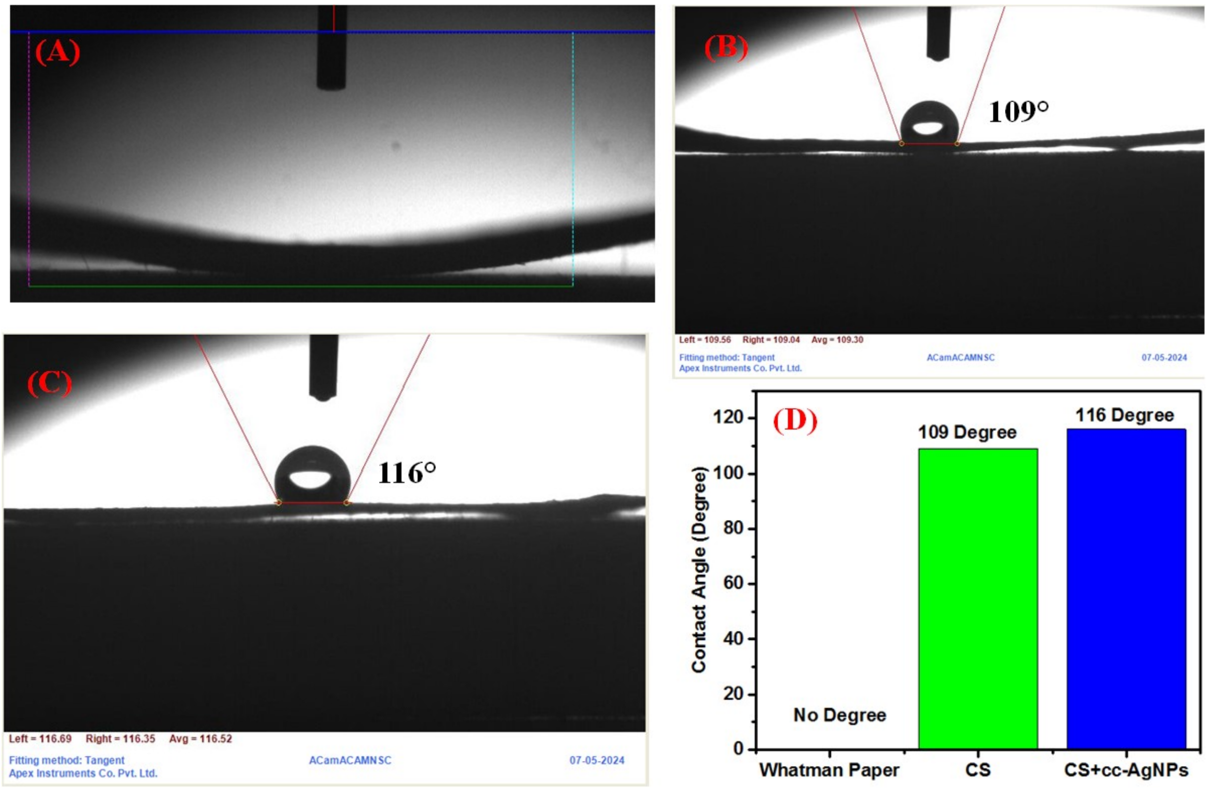

Fig. S1: (A) Contact angle measurements on Whatman paper, (B) CS film on Whatman paper, (C) CS+cc-AgNPs film on Whatman paper.

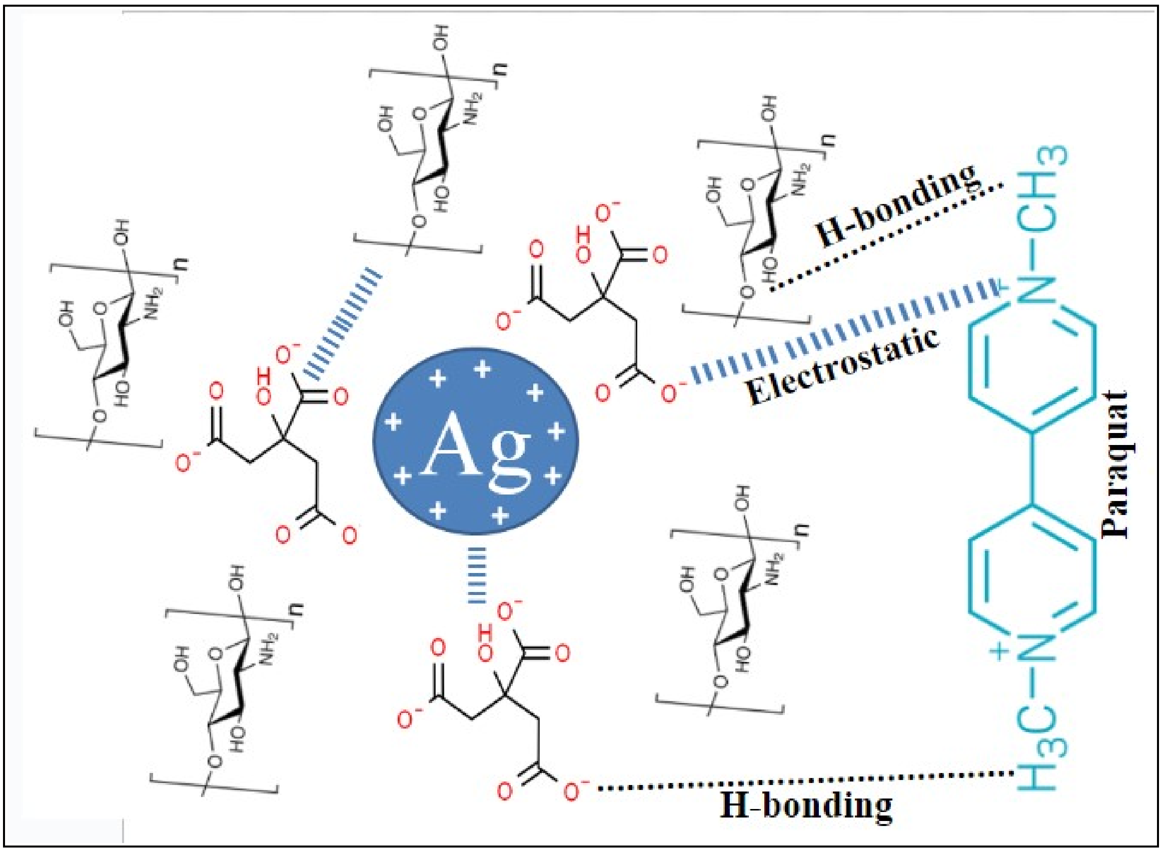

Fig. S2: Schematic diagram for sensing mechanism of paraquat pesticide.

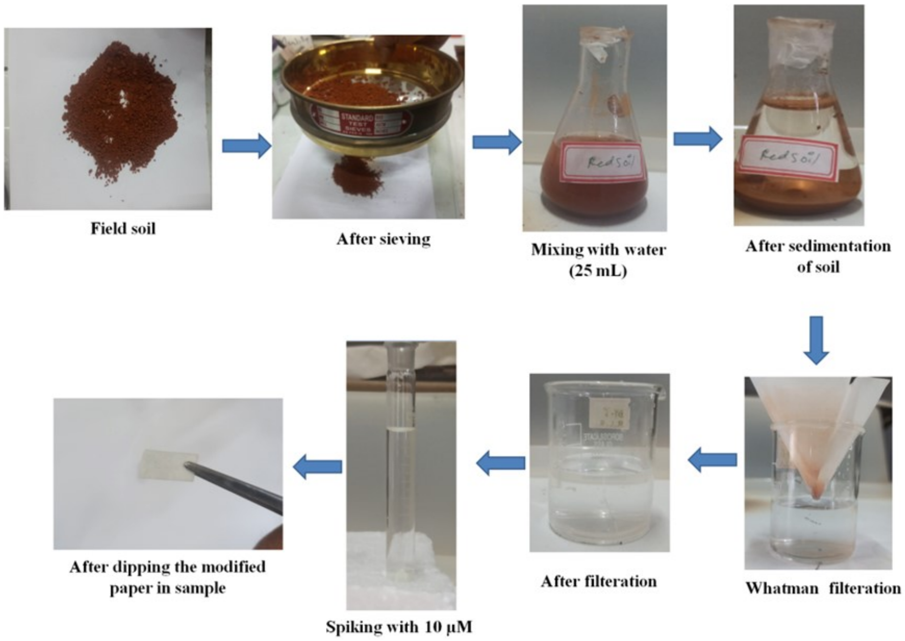

Fig. S3: Scheme for preparing soil samples
